# Oxymatrine and Cisplatin Synergistically Enhance Anti-tumor Immunity of CD8^+^ T Cells in Non-small Cell Lung Cancer

**DOI:** 10.3389/fonc.2018.00631

**Published:** 2018-12-18

**Authors:** Jin Ye, Man-Man Zou, Pei Li, Xi-Jun Lin, Qi-Wei Jiang, Yang Yang, Jia-Rong Huang, Meng-Ling Yuan, Zi-Hao Xing, Meng-Ning Wei, Yao Li, Zhi Shi, Hui Liu

**Affiliations:** ^1^Department of Otolaryngology-Head and Neck Surgery, The Third Affiliated Hospital, Sun Yat-sen University, Guangzhou, China; ^2^Division of Pulmonary and Critical Care, Department of Internal Medicine, The Third Affiliated Hospital, Sun Yat-sen University, Guangzhou, China; ^3^Guangdong Provincial Key Laboratory of Bioengineering Medicine, Department of Cell Biology & Institute of Biomedicine, National Engineering Research Center of Genetic Medicine, College of Life Science and Technology, Jinan University, Guangzhou, China

**Keywords:** oxymatrine, cisplatin, CD8^+^ T cells, anti-tumor immunity, NSCLC

## Abstract

Oxymatrine (OMT) has shown broad antitumor activities for the treatment of several types of cancers. However, little is known about its effect on anti-tumor immunity. Combination therapy is a potentially promising strategy of cancer to enhance anticancer activity, overcome drug resistance, and lower treatment failure rate. In the present study, we demonstrated that the combination of OMT with cisplatin (DDP) synergistically inhibited non-small cell lung cancer (NSCLC) cells growth when co-cultured with peripheral blood mononuclear cells *in vitro*. Furthermore, the combination of OMT with DDP significantly inhibited the growth of Lewis lung cancer (LLC) mouse xenograft tumors. Flow cytometry analysis revealed that OMT and DDP synergistically increase the CD8^+^/ regulatory T cells ratio and enhanced more CD8^+^ T cells secreted cytokines of IFN-γ, TNF-α, and IL-2 *in vivo*. Mechanistically, upregulation of *miR-155* and downregulation of suppressor of cytokine signaling-1 (*SOCS1*) were confirmed as a target signaling pathway to positively regulate the anti-tumor response of CD8^+^ T cells. Overall, OMT in combination with DDP showed outstanding synergistic anti-tumor immunity, suggesting that this beneficial combination may offer a potential immunotherapy for NSCLC patients.

## Introduction

Lung cancer is the leading cause of cancer-related death worldwide (1), and non-small cell lung cancer (NSCLC) accounts for approximately 85% of the whole lung cancer cases (2). Despite years of researches for early diagnosis and standard treatment, the prognosis for patients with lung cancer remains dismal, and 5-year survival rate remains < 15% (3–6). T cell–mediated anti-tumor immunotherapy emerges as a promising treatment for human malignancies, in which CD8^+^ T cells [cytotoxic T lymphocytes (CTLs)] represent a major of the cell-mediated anti-tumor response via providing host immune protection against intracellular pathogens and cancers (7, 8). However, progressive tumors can escape immune recognition and attack by smartly establishing an immune tolerance involving immunosuppressive T lymphocytes (9, 10). In particular, regulatory T cells (T_reg_) are proposed as key components of the immune suppressive tumor microenvironment with strong suppressive capacities toward CD4^+^ and CD8^+^ T lymphocytes, B cells, and dendritic cells etc. (11). Further, Baras et al. demonstrated that the CD8^+^/T_reg_ ratio in tumor infiltrating lymphocytes (TIL) densities rather than the two independent parameters was significantly associated with cisplatin-based neoadjuvant chemotherapy (12). MicroRNAs (miRNAs) have been confirmed as global regulators of gene expression programs that regulate specific target genes at the post-transcriptional level (13). Some of them have been identified as targets for anti-cancer therapeutics (14), and effects on tumor-infiltrating immune cells has become a hot spot besides their functions in cancer cells recent years (15). *miR-155* is an ancient regulator of the immune system (16). Elegant et al. demonstrated that *miR-155* was required for CD8^+^ T cell responses in defending against infection and cancer by silencing suppressor of cytokine signaling-1 (*SOCS1*) (17, 18). Initial evidence has also unraveled the crucial role of *miR-155* in dendritic cells functions in multiple types of cancers (19, 20). Altogether, these studies suggested the pivotal functions of *miR-155* in various T cell subsets as they respond to solid tumors.

Cisplatin (DDP)-based doublet remains the foundation of treatment for the patients with NSCLC in the modern era (21). The resistance of NSCLC cells to DDP is also an emergent problem, therefore developing more effective strategies for the treatment of NSCLC is urgently required. Combination chemotherapy is identified as a potentially promising approach to enhance anticancer activity, overcome drug resistance, and lower treatment failure rate (22, 23). Oxymatrine (OMT) is a main alkaloid extracted from roots of Sophora species with a broad range of bioactivities. Especially, extensive researches have reported that OMT have anticancer effects by inducing cell cycle arrest, apoptosis and inhibition of angiogenesis in various cancer cells *in vitro* and *in vivo* (24). In the previous studies, immunoregulatory effects of OMT on hepatitis B of mice, rheumatoid arthritis in rats and mastitis in mice have been confirmed (25–27). Considering the extensive effects of OMT, we investigate the effect of OMT in combination with DDP on anti-tumor immunity in NSCLC and elucidate the potential mechanism.

## Materials and Methods

### Cell Culture and Reagents

Human A549 NSCLC cell line and mouse Lewis lung cancer (LLC) cell line were cultured in Dulbecco' s modified Eagle's medium (DMEM) with 10% fetal bovine serum (FBS), penicillin (100 U/ml), and streptomycin (100 ng/ml) at 37°C with 5% CO_2_ in a humidified incubator. OMT and DDP were ordered from Dalian Meilun Biotechnology and Qilu Pharmaceutical, respectively. OMT and DDP were dissolved in phosphate-buffered saline (PBS) on stock concentration (1 M and 10 mM, respectively) and stored at −20°C. Other reagents were purchased from Shanghai Sangon Biotech unless otherwise noted.

### Cell Viability Assay

Freshly-isolated peripheral blood mononuclear cells (PBMCs) were suspended in DMED culture medium and seeded into a 96-well plate at a density of 1 × 10^4^ cells/well and treated with various concentrations of drugs in three parallel wells for 72 h. CCK-8 (Dojindo Molecular Technologies, Shanghai, China) was then added to each well according to the protocol of the manufacture. The absorbance was measured at wavelengths of 450 nm after incubation with CCK-8 solution at 37°C for 4 h. Cells viability assay of A549 and LLC cells were measured using methylthiazolyldiphenyl-tetrazolium bromide (MTT) (28). Briefly, tumor cells were distributed (5,000 cells/well) into 96-well plates containing agents at different concentrations. After 3 days, MTT was added to each well at a final concentration of 0.5 mg/ml. After incubation for 4 h, the medium and MTT solution were removed from each well, and formazan crystals were dissolved in 100 μl of DMSO. Absorbance was measured at wavelengths of 570 nm. All absorbance was detected by Multiscan Spectrum (Thermo Fisher). The concentrations required to inhibit growth by 50% (IC_50_) were calculated from survival curves using the Bliss method (29). Studies relative to human in this article were approved by the ethics committee of the Third Affiliated Hospital, Sun Yat-sen University (Approval No: [2014]2-17).

### Tumor Cells/PBMCs Co-culture

After adherence of tumor cells into 6-well plates (target cells, 4 × 10^5^ cells/well), a certain amount of PBMCs (effector cells) suspended in the appropriate DMEM pulsed with 10% FBS were added. Four ratios of effector cells to target cells, 0:1, 2:1, 4:1, and 6:1 were designed. After treated with OMT and DDP alone or combination, target cells (tumor cells) and effector cells (PBMCs) were co-cultured for 24 h at 37°C in 5% CO_2_. The cellular remaining viable tumor cells were photographed under microscope (OLYMPUS IX71) and quantified, respectively.

### Mice Xenograft Tumor Assay

Age-suitable C57BL/6 female mice were obtained from Vital River Laboratory Animal Technology (Beijing), and all mice have been maintained with sterilized food and water. All animal experimental procedures were approved by the Institutional Animal Care and Use Committee of Sun Yat-sen University (Approval No: IACUC-DB-17-0502). Briefly, female C57BL/6 mice within 6 weeks old and 20 g weight were used for each group. Each mouse was injected subcutaneously with LLC cells (2 × 10^6^ in 100 μl of PBS) in right scapular region. When the subcutaneous tumors were approximately 0.3 × 0.3 cm^2^ (two perpendicular diameters) in size, mice were randomized into four groups. Mice were injected intraperitoneally with vehicle alone (0.9% saline), OMT alone (100 mg/kg body weight per day), DDP alone (2 mg/kg body weight every 2 day), or a combination of OMT and DDP (administration method is as same as the relevant single drug group). The body weights of mice and the two perpendicular diameters (A and B) of tumors were recorded every day. The tumor volumes (V) were calculated according to the formula:

$$\begin{array}{l} \text{V}=\pi /6{\left(1/2\left(\text{A}+\text{B}\right)\right)}^{3} \end{array}$$

The mice were anesthetized after the experiment, and tumors were excised from the mice and weighted. Heparin anticoagulated peripheral blood, spleens and tumors were collected for further use. The rate of inhibition (IR) was calculated according to the formula below:

IR = 1-Mean tumor weight of experimental group/Mean tumor weight of control group × 100%.

### Flow Cytometric Analysis

PBMCs and spleen lymphocytes were collected with Ficoll-diatrizoate (LTS1077, tbdscience, Tianjin) according to the protocol of the manufacture. For the separation of tumor infiltrating lymphocytes from LLC-bearing mice, xenograft tumors were mechanically disrupted into 1 mm^3^ pieces and digested chemically in 7 ml of dissociation medium (DMEM medium plus with 10% FBS, collagenase type IV (5 mg/ml), DNase I (1 mg/ml), and hyaluronidase (1 mg/ml) for 30 min at 37°C followed by filtration through a 70 μm cell strainer (NEST Biotechnology, Wuxi). Dissociated tumor cells were washed twice by PBS. Erythrocytes were lysed by red blood cell lysing buffer (BD Pharmigen) if necessary. The following antibodies were used for staining: Fc block (anti-CD16/32, Cat: 553142), CD3 APC-A750 (Cat: 557596), CD8a BV510 (Cat: 563068), CD4a FITC (Cat: 553046), Foxp3 PE (Cat: 563101), CD45 Percp-cy5.5 (Cat: 550994), IFN-γ FITC (Cat: 554411), TNF-α (Cat: 554420), anti-IL-2 (eBioscience, Cat: 12-7021-81), PE Rat IgG2a, κ Isotype Control (Cat: 553930), FITC Rat IgG1, κ Isotype Control (Cat: 554684), APC Rat IgG1, κ Isotype Control (Cat: 554686), and Rat IgG2b kappa Isotype Control (eBioscience, Cat: 12-4031-82). As regards the concentrations of antibodies, 2 μl/test was used in PBMCs and spleen lymphocytes samples and 3 μl/test in TIL flow cytometry. All antibodies were purchased from BD Pharmigen unless otherwise noted. Briefly, all samples were block with anti-CD16/32 for 20 min on room temperature and then stained with appropriate antibodies for 30 min on ice. Anti-mouse FoxP3 staining (eBioscience, Cat: 00-5523) was used for intracellular staining according to the manufacturer's instructions. For intracellular staining of IFN-γ, TNF-α, and IL-2, single-cell suspensions were incubated at 37°C for 5 h in the presence of Cell Stimulation Cocktail (eBioscience, Cat: 85-00-4975-93) according to the manufacturer's protocol. Zombie Violet™ Fixable Viability Kit (Biolegend, Cat: 423113) was required to distinguish live/dead cells in tumor flow cytometry. Appropriate isotype control antibodies were used to determine the gating strategies.

### CD8^+^ T Cell Isolation

Freshly-separated single-cells of splenocytes were obtained according to the procedure above. For splenocytes CD8^+^ T cells isolation, CD8^+^ T cells were sorted by MACS (Miltenyi, Bergisch Gladbach, Germany) as described in the manufacturer's protocols. The purity of CD8^+^ T cells was >95%, confirmed by flow cytometry.

### Reverse Transcription Quantitative PCR

Total RNAs were extracted using RNeasy Mini Kit (Qiagen, Duesseldorf, Germany) in accordance with the manufacturer's instructions. We used 0.1 μg total RNAs as the template to synthesize cDNA via reverse transcription reaction through GoScript™ Reverse Transcription System kit (Promega) according to the manufacturer's instructions. For miRNA detection, equal RNA from each sample was reverse-transcribed to cDNA by means of specific miRNA stem-loop primers. Subsequently, quantitative real-time polymerase chain reactions were ran on Roche-LightCycler-480 by LightCycler 480 SYBR Green I Master. β*-actin* and *U6* were used as internal normalization controls. All assays were performed following the manufacturer's instructions. All sequences of primers listed in Table 1 were synthesized by Sangon Biotech (Shanghai, China). The thermal cycling conditions include 5 min at 95°C, and 40 cycles of 10 s at 95°C and 20 s at 55°C. Samples were run in triplicate and differences in gene expression were calculated using the 2^−cycle threshold^ method (30, 31).

**Table 1:** The primer sequences for real time PCR (mouse).

***miR-155* (RT)**	**5^′^-GTCGTATCCAGTGCAGGGTCCGAGGTATTCGCACTGGATACGACACCCCT-3^′^**
***miR-155* (F)**	**5^′^-GTCGTATCCAGTGCAGGGTCCGAGGTATTCGCACTGGATACGACACCCCT-3^′^**
***miR-155* (R)**	**5^′^-GTCGTATCCAGTGCAGGGTCCGAGGTATTCGCACTGGATACGACAAAATA-3^′^**
***U6* (RT)**	**5^′^-GTCGTATCCAGTGCAGGGTCCGAGGTATTCGCACTGGATACGACAAAATA-3**
***U6* (F)**	**5^′^-GCGCGTCGTGAAGCGTTC-3**
***U6* (R)**	**5^′^-GTGCAGGGTCCGAGGT-3**
***SOCS1* (F)**	**5^′^-CTGCGGCTTCTATTGGGGAC-3**
***SOCS1* (R)**	**5^′^-AAAAGGCAGTCGAAGGTCTCG-3**
**β-actin (F)**	**5^′^-CCTTCTTGGGTATGGAATCCTG-3**
**β-actin (R)**	**5^′^-CAATGCCTGGGTACATGGTG-3**

### Statistical Analysis

All results were presented as mean ± standard deviation (SD). Comparisons between the treated and untreated groups were performed with Student's *t*-test. All data were analyzed using GraphPad Prism 5 and a values of *P* < 0.05 was set statistically significant.

## Results

### OMT and DDP Synergistically Inhibit the Growth of NSCLC Cells Co-cultured With PBMCs *in vitro*

**Figure 1:**
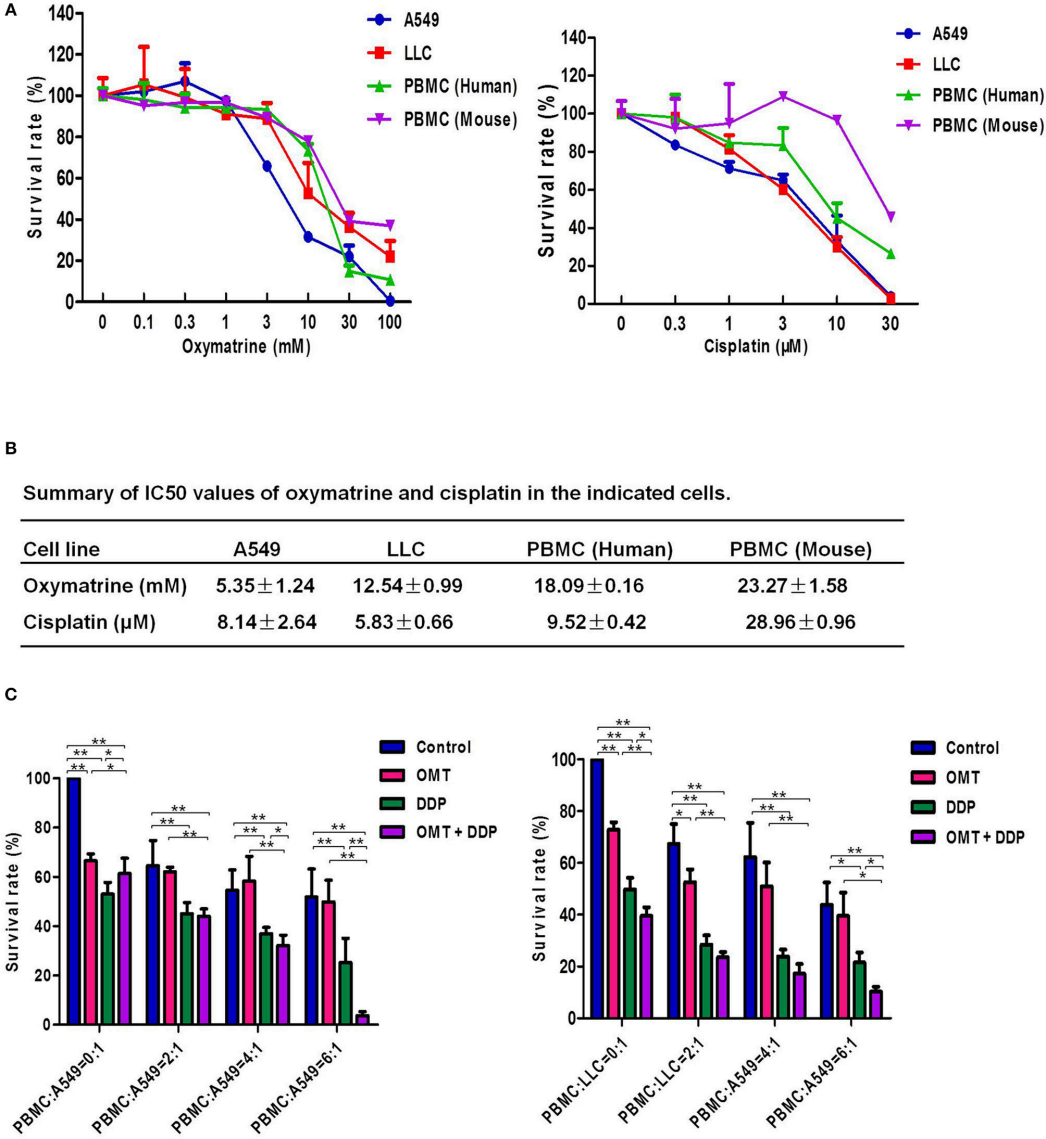
OMT and DDP synergistically inhibit the growth of NSCLC cells co-cultured with PBMCs *in vitro*. Cells were treated with the indicated concentrations of OMT or DDP for 72 h, and cell survival was determined by MTT or CCK-8 assay and summary survival curves **(A)** and IC_50_ values in the indicated cells **(B)** were shown. Co-cultured NSCLC cells (target cells) with PBMCs (effect cells) at ratios of 1:0, 1:2, 1:4, and 1:6 were treated with OMT (3 mM) and DDP (2 μM) alone or combination for 24 h. Quantified results were shown in **(C)**. The values presented are the means ± SD for each group. *P < 0.05 and **P < 0.01 *vs*. corresponding control.

In the present study, we firstly investigate the effects of OMT and DDP on NSCLC cells and PBMCs. Cell survival was assessed by MTT assay. As shown in Figures 1A,B, the survival of all used cells was decreased in a dose-dependent manner *in vitro* after OMT or DDP treatment. OMT and DDP exhibited significant cytotoxicity against A549 and LLC cells, but weaker cytotoxicity against human and mice PBMCs. To assess the anti-tumor effects of OMT and DDP on growth of NSCLC cells in the presence of PBMCs, co-cultured NSCLC cells (target cells) with PBMCs (effect cells) at ratios of 1:0, 1:2, 1:4, and 1:6 were treated with OMT and DDP alone or combination. As showed in Figure 1C, after co-treatment with OMT and DDP, the survival of cancer cells were significantly reduced in comparison with OMT or DDP alone without PBMCs. Strikingly, the growth of tumor cells were more potently inhibited by OMT or (and) DDP administration when co-cultivated in combination with PBMCs at all target/effector cells ratio, and especially the ratio of target/effect cells at 1:6 exhibited most effective inhibition. These results suggest that OMT and DDP synergistically inhibit the growth of lung cancer cells when co-cultured with PBMCs *in vitro*.

### OMT and DDP Synergistically Inhibit the NSCLC Xenografts Growth *in vivo*

**Figure 2:**
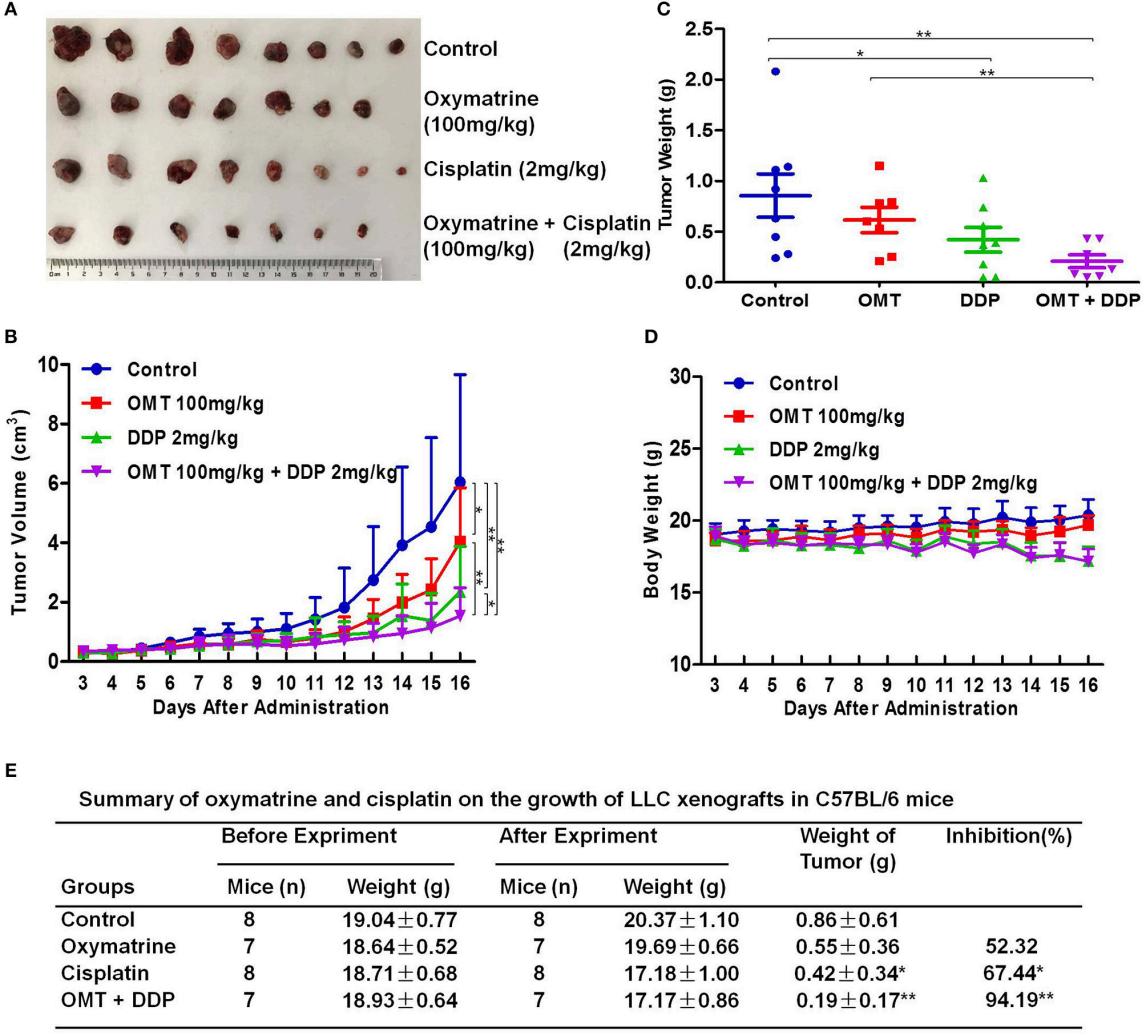
OMT and DDP synergistically inhibit NSCLC xenografts growth *in vivo*. Each mouse was injected subcutaneously with LLC cells (2 × 10^6^ in 100 μl of PBS) in right scapular region. When the subcutaneous tumors were approximately 0.3 × 0.3 cm^2^ (two perpendicular diameters) in size, mice were randomized into four groups, and were injected intraperitoneally with vehicle alone (0.9% saline), OMT alone (100 mg/kg body weight per day), DDP alone (2 mg/kg body weight every 2 day), or a combination of OMT and DDP (administration method is as same as the relevant single drug group). The body weights and tumor volumes of mice were recorded. The mice were anesthetized after experiment, and tumors were excised from the mice and weighted. The original tumors **(A)**, tumor volumes **(B)**, tumor weights **(C)**, body weights **(D)**, and summary data **(E)** were shown. The values presented are the means ± SD for each group. *P < 0.05 and **P < 0.01 vs. corresponding control.

To examine the synergistic anti-tumor effects of OMT and DDP *in vivo*, we generated the xenograft tumor models by transplanting LLC cells into C57BL/6 mice. As shown in Figures 2A–C, compared with OMT or DDP alone treatment, co-treatment OMT with DDP significantly inhibited the growth of subcutaneous tumors by diminishing the volume and weight of tumors. The inhibition rate of tumor growth in the co-treatment group reached 94.19%, which was obviously higher than that in either single treatment group (Figure 2E). In addition, mice body weights in DDP alone or co-treatment groups were lower than those of control group (Figure 2D). These data suggest that OMT and DDP can synergistically inhibit the NSCLC xenograft growth *in vivo*.

### OMT and DDP Synergistically Increase the CD8^+^/T_reg_ Ratio *in vivo*

**Figure 3:**
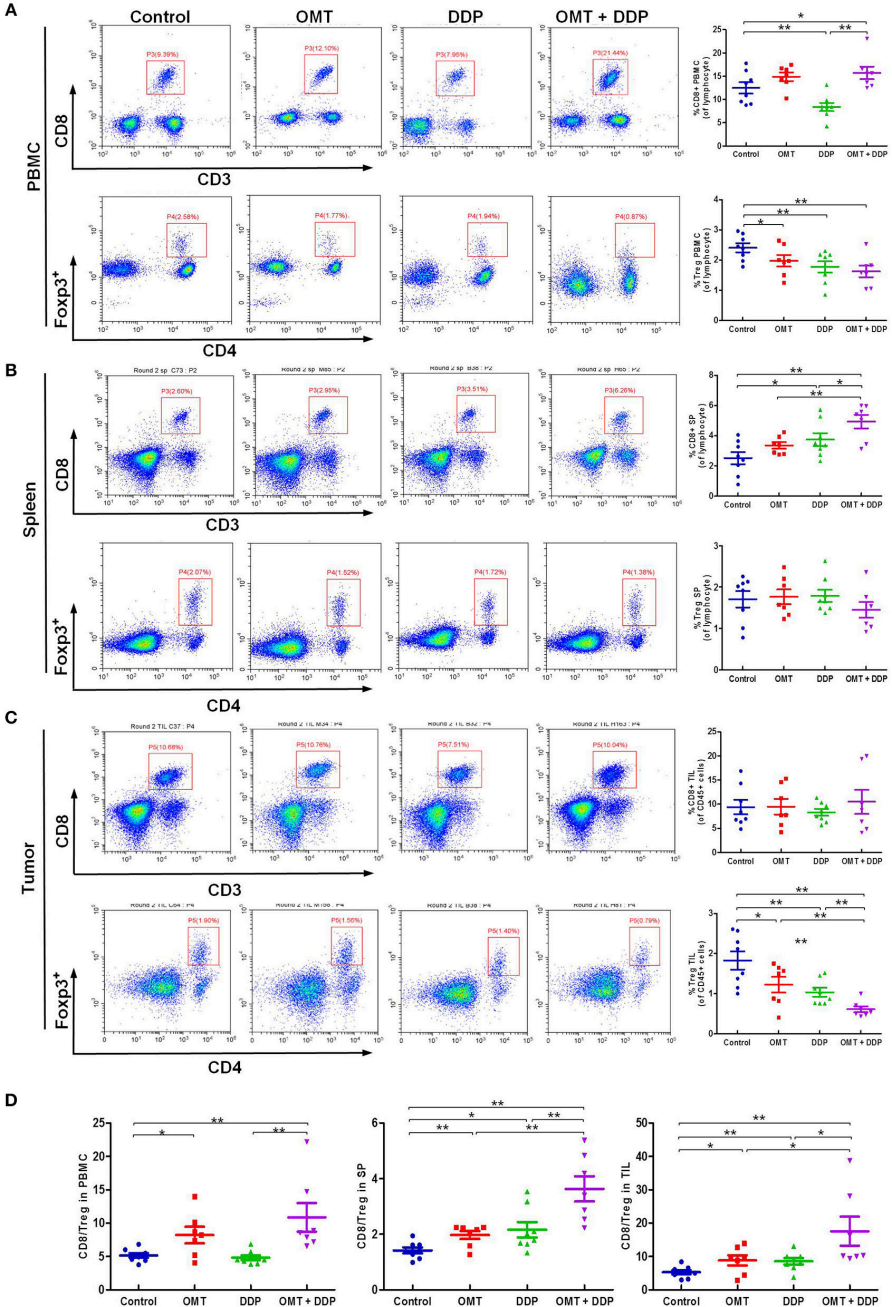
OMT and DDP synergistically increase the CD8^+^/T_reg_ ratio *in vivo*. Isolated PBMCs, spleen lymphocytes and tumor infiltrating lymphocytes were stained with indicated antibodies and analyzed by flow cytometry. Representative flow plots and quantified results of CD8^+^ T cells and T_reg_ cells in PBMCs **(A)**, spleen lymphocytes **(B)**, and tumor infiltrating lymphocytes **(C)** were shown. The CD8^+^/T_reg_ ratios were quantified **(D)**. *P < 0.05 and **P < 0.01 vs. corresponding control.

The interaction of immune system in malignant diseases is heralded as one of the most important advances in oncology. We speculated that heightened tumor regression after OMT and DDP treatments may be caused by strong anti-tumor immunity. The cytotoxic T lymphocytes (CTLs, also CD8^+^ T cells, marked as CD3^+^CD8^+^ T cells) are pivotal immune cells directed against tumor cells susceptible to cell lysis, but CD4^+^Foxp3^+^ regulatory T cells (T_reg_) disturb antitumor immunity by suppressing the activities of effector T cells. Our flow cytometry data revealed that compared with OMT or DDP alone treatment, co-treatment OMT with DDP significantly increased CD8^+^ T cells percentage in PBMCs and spleen lymphocytes, and decreased T_regs_ cells percentage in PBMCs and tumor infiltrating lymphocytes (Figures 3A–C). Furthermore, compared with OMT or DDP alone treatment, co-treatment OMT with DDP significantly increased the CD8^+^/T_reg_ ratio in PBMCs, spleen lymphocytes and tumor infiltrating lymphocytes (Figure 3D). These results indicate that OMT and DDP can synergistically increase the CD8^+^/T_reg_ ratio *in vivo*.

### OMT and DDP Synergistically Enhance CD8^+^ T Cells Anti-tumor Immune Response

**Figure 4:**
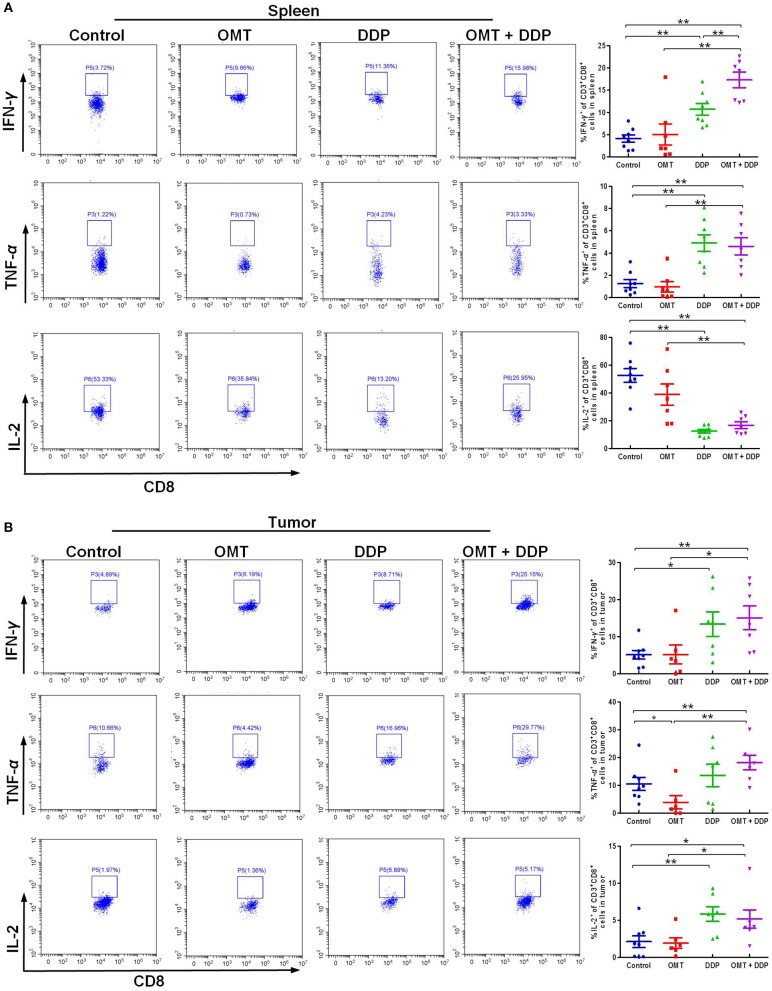
OMT and DDP synergistically enhance CD8^+^ T cells anti-tumor immune response. Spleen lymphocytes and tumor infiltrating lymphocytes were isolated, and intracellular IFN-γ, TNF-α, and IL-2 were determined by flow cytometry. Representative flow plots and quantified results of intracellular IFN-γ, TNF-α, and IL-2 expression in CD8^+^ T cells of spleen lymphocytes **(A)** and tumor infiltrating lymphocytes **(B)** were shown. *P < 0.05 and **P < 0.01 vs. corresponding control.

We further evaluated the immune status of CD8^+^ T cells in mice bearing LLC, since CD8^+^ T cells play a pivotal role in anti-tumor immunity. As shown in Figures 4A,B, compared with OMT or DDP alone treatment, co-treatment OMT with DDP significantly induced the increased intracellular IFN-γ and TNF-α and the decreased intracellular IL-2 in spleen lymphocytes, and the increased intracellular IFN-γ, TNF-α, and IL-2 in tumor infiltrating lymphocytes, suggesting that OMT and DDP synergistically enhance CD8^+^ T cells anti-tumor immune response.

### OMT and DDP Synergistically Upregulate *miR-155* and Downregulate *SOCS1* Expressions in Splenic CD8^+^ T Cells

**Figure 5:**
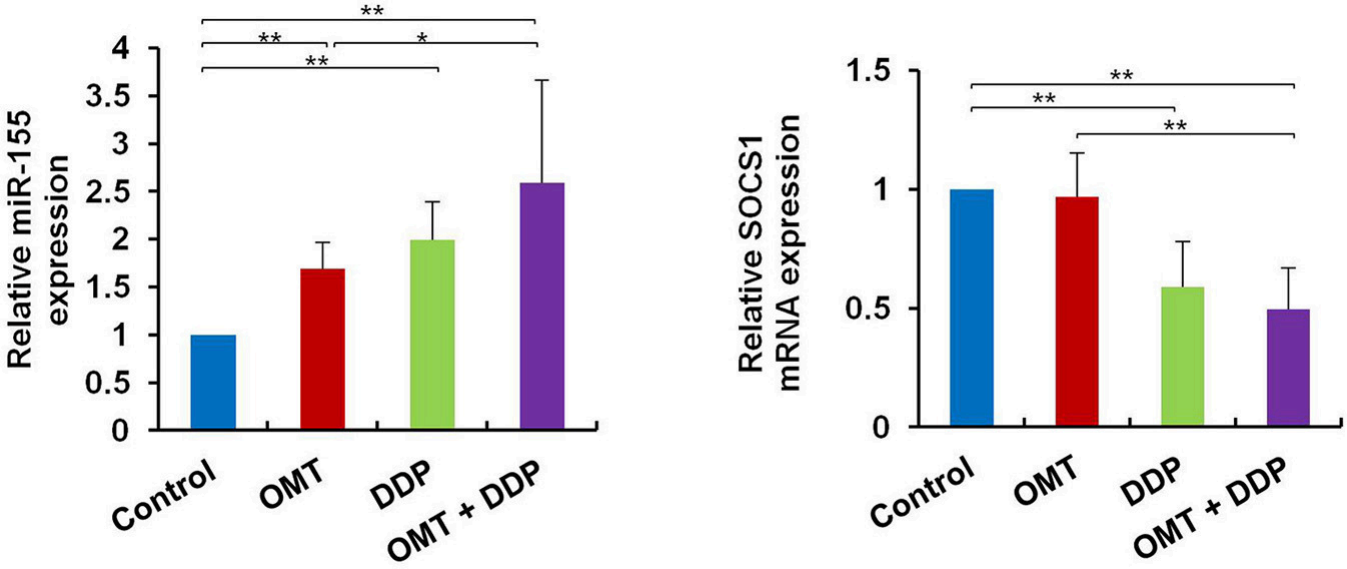
OMT and DDP synergistically upregulate *miR-155* and downregulate *SOCS1* expressions in splenic CD8^+^ T cells. Splenocytes CD8^+^ T cells were separated by magnetic bead from MACS and the total RNAs were extracted immediately. Expression of *miR-155* and *SOCS1* in splenic CD8^+^ T cells were determined by RT-qPCR. *U6* and β-actin were used as the normal controls. Data shown are representative of three independent experiments. *P < 0.05 and **P < 0.01 vs. corresponding control.

*MiR-155* plays a key role in tumor immune response by targeting *SOCS1* (16). We detected *miR-155* and *SOCS1* expressions in splenic CD8^+^ T cells. As shown in Figure 5, compared with OMT or DDP alone treatment, co-treatment with OMT and DDP significantly upregulated *miR-155* and downregulate *SOCS1* expressions in splenic CD8^+^ T cells.

## Discussion

Natural products play an important role in the prevention and treatment of cancer and other disease in the world (32, 33). Our study clearly indicates that the combination of OMT and DDP synergistically enhanced NSCLC cells growth inhibition, CD8^+^/_Treg_ ratio and CD8^+^ T cells anti-tumor immune response with the upregulation of *miR-155* and the silence of *SOCS1*. It has been reported that cancer immunotherapy has been a hot spot in the treatment of NSCLC (34). T_reg_ cells are highly immune suppressive cells and play central roles in the maintenance of self-tolerance and immune homeostasis (35). It can inhibit anti-tumor immunity in NSCLC by suppressing effector T cells directly by cell interaction or indirectly via the secretion of soluble factor-mediated suppression (36). We previously have reported that higher T_reg_/CD8^+^ ratio in tumor was an independent factor for poor response to platinum-based chemotherapy, but CD8^+^ and T_reg_ tumor infiltrating lymphocytes was not correlated with any clinicopathological features in advanced NSCLC patients (37). Current findings on the model of mice bearing LLC have suggested that co-treatment OMT with DDP significantly enhanced the CD8^+^/T_reg_ ratio in comparison with single agent groups, which is also in agreement with other clinical evidence that decreased CD8^+^/T_reg_ ratio among tumor infiltrating lymphocytes are correlated with poor prognosis in various types of human cancers (38–40). However, there are differences between the results of the present study and a report by Zhang et al. which demonstrated that higher ratio of CD8^+^/ T_reg_ was significantly associated with poor overall survival and progression-free survival in early nasopharyngeal carcinoma stage patients (41). Different chemotherapeutic regimens and tumor context may contribute to these differences.

It is well-known that CD8^+^ effector T cells have a critical role in elimination of tumors. Previous studies showed that IFN-γ, TNF-α, and IL-2-expressing CD8^+^ T cells are instrumental in anti-tumor immune response (42). IFN-γ-expressing T cells are essential in repressing tumor growth which promotes host responses to tumors. Moreover, IFN-γ can execute direct anti-proliferative, pro-apoptotic and anti-angiogenesis actions on various tumor cells (43). TNF-α is another multifunctional cytokine, which mediates anticancer adaptive immune response. In the report of Ando et al. TNF-α might be an effective therapy in some cases of NSCLC that have acquired resistance to gefitinib (44). IL-2 acts crossroads functions in activation and cell growth of T and NK cells and it can promote CD8^+^ T cells and natural killer cells cytolytic activities in response to antigen (45). In lung cancer patients, IL-2 treatment reverses CD8^+^ T cells exhaustion and markedly increases Granzyme B and IFN-γ in malignant pleural effusion. Our study indicated that OMT in combination with DDP significantly upregulated the production of IFN-γ and TNF-α in CD8^+^ T cells compared with the single agent both in the splenocytes and tumor infiltrating lymphocytes. Nevertheless, expression of IL-2 is declined in splenocytes and increased in tumor infiltrating lymphocytes, inversely. These differences indicated the complexities of the effects of chemotherapeutic drugs in different immune organs. Since IL-2 is essential for the development and maintenance of T_reg_ (45), declined IL-2 secretion may be able to decrease the immune suppressive T_reg_. This may be another manner to enhance CD8^+^ T cells anti-tumor response to a certain extent.

One particular miRNA, *miR-155*, has emerged as a central regulator in immune homeostasis and antitumor immunity recent years (16, 46). *MiR-155* silencing promotes solid tumor growth through increasing the recruitment and functions of myeloid-derived suppressor cells in tumor microenvironment (47). Strikingly, *miR-155* can augments effector CD8^+^ T-cell anti-tumor immunity against viruses and cancer (17, 18, 48, 49). In detail, *miR-155* overexpression and silence of its target *SOCS1* in CD8^+^ T cells enhanced the antitumor response and augmented tumor destruction (17). According to Ji et al.'s report, *miR-155* restrained the expression of *SOCS1*, one of the negative regulators of signal transducer and activator of transcription 5 (*STAT5a*), and constitutively active *STAT5a* recapitulated the survival advantages conferred by miR-155 (18). In addition, it is reported that *miR-155* shapes cytokine signaling via downregulation of *SOCS1* in T_reg_ subsets. Consistently, these findings consider *miRNA-155* and its target *SOCS1* as key regulators of effector CD8^+^ T cells that can be modulated to potentiate immunotherapies for cancers. In our study, increased *miR-155* and decreased *SOCS1* expressions in splenic CD8^+^ T cells are much agreement with the aforementioned investigations, which demonstrated that co-treatment OMT with DDP can enhanced antitumor immunity via *miR-155-SOCS1* signaling pathway in mice bearing LLC tumor. Further researches need to elucidate the effects of “loss or gain” functions of *miR-155* gene in our mice NSCLC model when OMT co-treatment with DDP. Moreover, antitumor immunity is most complicated involved in effector and immunosuppressive networks in the tumor microenvironment. In addition to CD8^+^ T cells and T_regs_, dendritic cells, natural killer cells, suppressive dysfunctional dendritic cells and macrophagocytes, these are essential immunogenic elements to skew the balance of pro- and anti-tumor forces toward tumor-specific immunity (50). Their immunomodulated functions in our present study need to be further investigated.

Collectively, the present study offers the first evidence that OMT and DDP synergistically inhibit the growth of NSCLC cells when co-culture with PBMCs *in vitro*. Further *in vivo* studies provide strong evidence that combinational treatment OMT with DDP shows outstanding synergistic anticancer effect by tipping a favor anti-tumor immunity, suggesting this beneficial combination may offer a promising treatment option for NSCLC patients.

## Ethics Statement

Studies relative to human in this article were approved by the ethics committee of the Third Affiliated Hospital, Sun Yat-sen University (Approval No: [2014]2-17). All animal experiments were performed strictly in accordance with the Guidelines for the Care and Use of Laboratory Animals (No. 55 issued by Ministry of Health, China on January 25, 1998), and experimental procedure were approved by the Institutional Animal Care and Use Committee of Sun Yat-sen University (Approval No: IACUC-DB-17-0502).

## Author Contributions

JY, M-MZ, ZS, and HL designed the experiments, performed the experiments, analyzed the data, and wrote the paper. PL, X-JL, Q-WJ, YY, J-RH, M-LY, Z-HX, M-NW, and YL performed the experiments. All authors read and approved the final manuscript.

### Conflict of Interest Statement

The authors declare that the research was conducted in the absence of any commercial or financial relationships that could be construed as a potential conflict of interest.
